# Sample Shuttling Relaxometry of Contrast Agents: NMRD Profiles above 1 T with a Single Device

**DOI:** 10.1007/s00723-015-0751-7

**Published:** 2016-01-30

**Authors:** Yves Gossuin, Zeinab Serhan, Lydia Sandiford, Daniel Henrard, Thorsten Marquardsen, Rafael T. M. de Rosales, Dimitrios Sakellariou, Fabien Ferrage

**Affiliations:** Biomedical Physics Department, University of Mons, 24, Avenue du Champ de Mars, 7000 Mons, Belgium; Département de Chimie, École Normale Supérieure - PSL Research University, 24 rue Lhomond, 75005 Paris, France; Sorbonne Universités UPMC Univ Paris 06, LBM, 4 place Jussieu, 75005 Paris, France; UMR 7203 LBM, CNRS, 75005 Paris, France; Division of Imaging Sciences and Biomedical Engineering, St. Thomas’ Hospital, King’s College London, London, SE1 7EH UK; Bruker BioSpin GmbH, Silberstreifen 4, 76287 Rheinstetten, Germany; Laboratoire Structure et Dynamique par Résonance Magnétique, CEA Saclay, DSM, IRAMIS, UMR CEA/CNRS 3685, NIMBE, 91191 Gif-sur-Yvette Cedex, France

## Abstract

Nuclear magnetic relaxation dispersion (NMRD) profiles are essential tools to evaluate the efficiency and investigate the properties of magnetic compounds used as contrast agents for magnetic resonance imaging (MRI), namely gadolinium chelates and superparamagnetic iron oxide particles. These curves represent the evolution of proton relaxation rates with the magnetic field. NMRD profiles are unparalleled to probe extensively the spectral density function involved in the relaxation of water in the presence of the paramagnetic ion or the magnetic nanoparticles. This makes such profiles an excellent test of the adequacy of a theoretical relaxation model and allow for a predictive approach to the development and optimization of contrast agents. From a practical point of view they also allow to evaluate the efficiency of a contrast agent in a certain range of magnetic fields. Nowadays, these curves are recorded with commercial fast field cycling devices, often limited to a maximum Larmor frequency of 40 MHz (0.94 T). In this article, relaxation data were acquired on a wide range of magnetic fields, from 3.5 × 10^−4^ to 14 T, for a gadolinium-based contrast agent and for PEGylated iron oxide nanoparticles. We show that the low-field NMRD curves can be completed with high-field data obtained on a shuttle apparatus device using the superconductive magnet of a high-field spectrometer. This allows a better characterization of the contrast agents at relevant magnetic fields for clinical and preclinical MRI, but also refines the experimental data that could be used for the validation of relaxation models.

**Electronic supplementary material:**

The online version of this article (doi:10.1007/s00723-015-0751-7) contains supplementary material, which is available to authorized users.

## Introduction

Nuclear magnetic resonance (NMR) relaxometry consists in the measurement of the relaxation times (*T*_1_, *T*_2_) of a nucleus observable in NMR as a function of the magnetic field. The dependence of the relaxation rates (*R*_1_ = 1/*T*_1_ and *R*_2_ = 1/*T*_2_) with the magnetic field bears important information since it gives access to the spectral density and thus to the mechanism of relaxation [1, 2]. This allows one to probe the molecular dynamics of different systems such as proteins, polymers, and water trapped in porous systems. Relaxometry provides sufficiently extensive experimental datasets so that the relaxation mechanisms can be determined in yet poorly characterized systems containing magnetic entities. This is especially true for water proton relaxation induced by paramagnetic ions [3] and superparamagnetic particles used as contrast agents for magnetic resonance imaging [4]. The curves representing the evolution of relaxation rates with the magnetic field are called nuclear magnetic relaxation dispersion (NMRD) profiles. The term “dispersion” indicates that for many systems the rates decrease for increasing fields, which reflects the dispersion of the spectral density function for increasing Larmor frequencies. Experimentally the *R*_1_ NMRD curves are often measured with the fast field-cycling (FFC) technique, and can be complemented by conventional *R*_1_ measurements on NMR devices working at a single field. Recording *R*_2_ rates requires the application of a series of refocusing radiofrequency pulses, which is difficult on fast field-cycling systems. *R*_2_ NMRD profiles are thus obtained through measurements on a series of instruments, relaxometers and spectrometers. The use of high field spectrometers already allows to probe the high field region of the NMRD profiles [5–9]. However, it necessitates the access to numerous instruments and can be time consuming.

Using FFC, the sample is submitted to sudden changes of magnetic field from the polarization field B_*pol*_ to the relaxation field B_*rel*_, which causes relaxation, without using any excitation pulse. The detection is always done at the same field B_*det*_, whatever the relaxation field B_*rel*_ is. This allows to use a single probe tuned at the resonance frequency of the detection field B_*det*_, where a 90° pulse has to be used to record a free induction decay. In available commercial devices, called FFC-relaxometers, the change in magnetic field is caused by a change in the electrical current circulating in an electromagnet. The technique is demanding for both the magnet and its cooling system. Indeed the Joule effect is considerable and the heat produced at the magnet must be evacuated through an appropriate cooling system to ensure magnetic field stability. As a consequence, the maximum electromagnet field accessible is limited: magnetic fields can be as low as 0.23 mT but often limited to 1 T. However, the field change can be really fast (~1 ms) which allows the measurement of short *T*_1_. In order to reach higher fields, the sample shuttle technique can be used: it consists in moving the sample in different regions of the stray field of a strong superconducting magnet, usually the magnet of a commercial high-field NMR spectrometer. The motion of the shuttle can be driven by a pneumatic system [10–12] or a motorized apparatus [13, 14]. The speed at which the field can be changed is limited by the motion of the sample and thus slower than in FFC-relaxometers (~50–100 ms) so that the measurement of short *T*_1_ is challenging. However, the maximum magnetic field accessible is limited to the magnetic field of a high-field NMR magnet. In the case of contrast agents, this covers, in principle, all fields accessible to MRI. Excellent descriptions of the FFC techniques and of some applications can be found in the literature [15–17].

The development of the theories describing the relaxation induced by paramagnetic and superparamagnetic contrast agents is closely related to the measurement of NMRD profiles of aqueous suspensions of these magnetic systems. Indeed theses curves constitute unique experimental data to test the adequacy of the theoretical models through a simple fitting of the NMRD profile. From a fundamental point of view, *T*_1_ data at fields larger than 1 T can therefore be necessary to test/develop relaxation models for magnetic contrast agents.

From a more practical point of view, NMRD profiles of contrast agents also provide at a glance their relaxation efficiency at different Larmor frequencies, which is valuable for MRI at a given field. However, most new MRI systems operate at 3 T, typical small animal MRI systems operate at 7 T while some new devices reach 21 T. Such fields are not accessible to commercial FFC equipments which are often limited to 1 T, while some recent hybrid systems using a superconducting magnet reach 3 T. In this communication, we show that both the low-field (obtained with a commercial device) and high-field NMRD profiles (recorded with a shuttle system) are necessary for the evaluation of contrast agents efficiency as well as for the development of relaxation models for magnetic contrast agents.

## Materials and methods

Oleylamine- and PEG-coated ultra small superparamagnetic iron oxide particles (USPIOs) were synthesized using a two-step reaction based on a modification of a recently published method [18]. First, 1.042 g Fe(acac)_3_ were added to 30 mL of oleylamine. The solution was gradually heated to 128 °C at a rate of 363.5 °C/h under a N_2_ flow followed by a temperature increase to 180 °C over a period of 1 h, and finally heating to 270 °C at a ramping rate of 396 °C/h after which the heating appliance was removed. The solution was left to cool to room temperature and the oleylamine-iron oxide nanoparticles precipitated upon the addition of 30 mL of ethanol, followed by centrifugation at 9000*g* for 4 min. The supernatant was discarded and the process repeated with another 35 mL of ethanol, then a further 56 mL. The resulting particles had a core of 5.2 ± 0.7 nm, based on the statistical analysis of 100 particles observed by transmission electron microscopy (TEM). The process of functionalization with PEG(5)-BP [polyethylene glycol (5 kDa)-bisphosphonate] allowed for high yields to be reached in a short time and at room temperature. First, 1 mg oleylamine-coated USPIOs and 10 mg PEG(5)-BP were added to 1 mL of dichloromethane in an open glass vial, and the mixture was sonicated for ~15 min until the solvent had evaporated. 2 mL of water were added to the remaining residue resulting in a clear brown solution. The mixture was washed with 2 mL of hexane to remove the oleylamine, followed by removal of hexane by evaporation under a N_2_ flow. This process was repeated two more times. The final mixture was filtered through a 0.2 µm hydrophilic polytetrafluoroethylene filter, followed by several cycles of washing/concentrating using a Vivaspin two centrifugal filter (30 kDa molecular weight cut off) using water to remove excess PEG(5)-BP leaving an amber dispersion of PEG(5)-BP-USPIOs. After PEGylation, there was no significant difference in the core size of the PEG(5)-BP-USPIOs (5.2 ± 0.6 nm based on the statistical analysis of 100 particles), and the hydrodynamic diameter, measured using dynamic light scattering (DLS), was 30 ± 9 nm.

GadoSpin™ P was purchased from Miltenyi Biotec GmbH (Germany). It is a polymeric gadolinium-based contrast agent (intended for MRI of small animals) containing several gadolinium chelates (Gd-DTPA-pentaamide) bound to a polymer backbone. The synthesis protocol is described in [19]. The molecular weight of the molecule is about 200 kDa. The lyophilized compound was reconstituted with 850 µL of physiological saline solution, which provided a final Gd^3+^ concentration of 20 mM. The stock solution was further diluted in the same buffer to reach a final Gd^3+^ concentration of 0.5 mM for sample shuttling measurements.

Low-field NMRD profiles (*T*_1_) of aqueous suspensions were measured from 0.015 to 40 MHz with a Spinmaster FFC relaxometer (STELAR, Mede, Italy) at 25 °C using 600 µL of suspensions in a dedicated NMR tube.

**Fig. 1:**
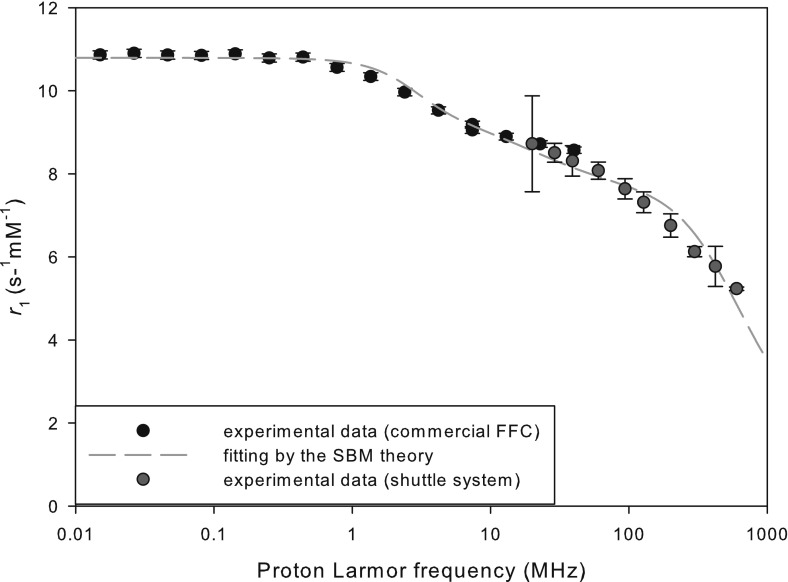
*T*_1_ NMRD profile of PEGylated iron oxide nanoparticles at 25 °C

**Fig. 2:**
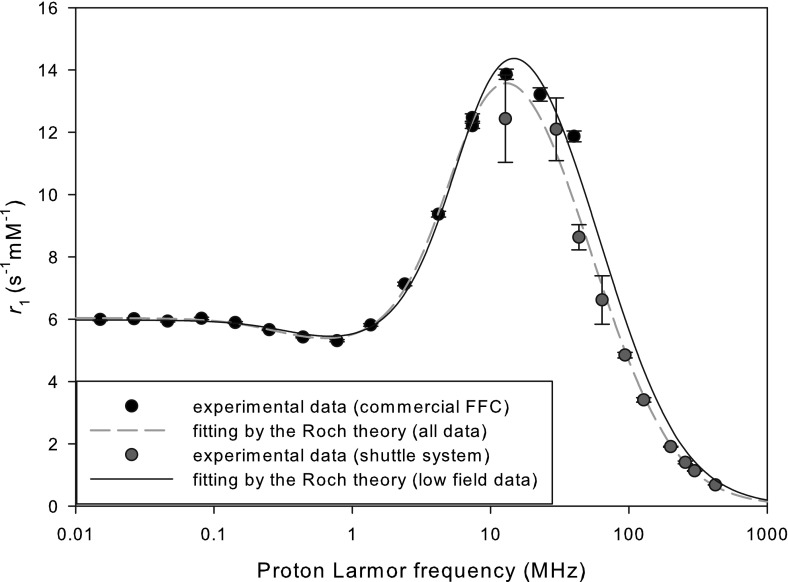
*T*_1_ NMRD profile of Gadospin™ at 25 °C

The high-field parts of the NMRD profiles were recorded on a Bruker Avance IIIHD 600 MHz spectrometer equipped with a pneumatic sample shuttle already described in detail [11, 12]. The magnetic field above the magnetic center was measured in steps of 1 mm using a homemade device with two calibrated triple-axes Hall probes (Senis) with a precision of 0.1 %. A CH3A10mE3D transducer was used for measurements from 0.05 to 2 T, while a 03A05F-A20T0K5Q transducer was used between 1 and 13 T. A systematic error of up to 3 % cannot be excluded and was reported on Figs. 1 and 2. The magnetic field for relaxation was constant with 1 % for all measurements carried out at each magnetic field (this corresponds to a displacement of the top position by less than 1 mm). The system includes a triple-resonance (^1^H, ^13^C, ^15^N) probe with a z-gradient. The low volume of the sample (100 μL) and the fact that detection on the proton channel is performed with the outer coil make this system mostly immune to radiation damping. Longitudinal relaxation rates at 14.1 T were measured using saturation recovery experiments. All other relaxation rates were measured with a sequence given as supporting information (Fig. S1). After a 5 s delay for polarization at 14.1 T, the longitudinal polarization is inverted every other scan before the transfer to the desired low-field spot, the sample shuttle is transferred back to 14.1 T after the relaxation delay for detection (see Fig. S1). The measured intensity can be fitted by a single exponential, which decays towards zero. In the case of the Gadospin study, two sub-spectra (with and without inversion at high field) were fitted independently, as the measurement of signal differences was challenging in the absence of lock (no D_2_O was added to the sample). All experiments were repeated three times, error bars represent the standard deviation of the three (six) fitted relaxation rates for USPIO (respectively, Gadospin) samples. The error was larger for low fields because of relaxation occurring during the transport of the sample, especially for small relaxation times. All exponential fits were carried with the *T*_1_/*T*_2_ module of the Topspin software.

The iron and gadolinium concentrations of the samples were determined by Inductively Coupled Plasma-Atomic Emission Spectrometry (ICP-AES) after microwave digestion in a mixture of nitric acid and hydrogen peroxide. All the relaxation data are presented as relaxivities *r*_1_, defined as the relaxation rate *R*_1_ = 1/*T*_1_ normalized by the iron (or gadolinium) concentration.

## Results and discussion

Figures 1 and 2, respectively, present the *T*_1_ NMRD profiles of PEG(5)-BP-USPIO particles and Gadospin™ paramagnetic contrast agent. For both compounds, the data obtained at high field with the shuttle system are in good agreement with the low-field data measured with the commercial FFC device. This can be verified in particular by the inspection of the data obtained on both equipments between 20 and 40 MHz. This zone is crucial since it allows for a direct comparison of the results obtained with the two techniques. The concentration used for high-field NMRDs was smaller than for low-field NMRDs since the shuttle times are rather large compared to the electronic switching times. As relaxation must be minimal during these time intervals, samples with longer relaxation times must be used with the shuttle device. It is worth noting that the 600 MHz measurement, obtained by a conventional saturation-recovery sequence in the normal configuration of the spectrometer, is clearly compatible with the data obtained at lower fields with the shuttle system.

The relaxation data of the superparamagnetic nanoparticles was fitted with the theory developed by Roch et al. [4] with a water diffusion coefficient at 25 °C of 2.3 × 10^−9^ m/s^2^. The fitted parameters are provided in Table 1.

**Table 1:** Parameters obtained from the fits of the NMRD profiles

Iron oxide nanoparticles	*M*_sat_ *(A/m)*	*R (nm)*	*τ*_*N*_ *(ns)*	*p*
All data	(189 ± 5) 10^3^	4.64 ± 0.2	1.16 ± 0.11	0.27 ± 0.5
Low field data	(205 ± 4) 10^3^	4.27 ± 0.09	0.89 ± 0.05	0.24 ± 0.5

Gadospin™	*τ*_*R*_ *(ns)*	*τ*_*M*_ *(µs)*	*τ*_*SO*_ *(ns)*	*τ*_*V*_ *(ps)*	*τ*_*SS*_ *(ps)*	*q*_*SS*_
	0.480 ± 0.024	2.46 ± 0.05	0.13 ± 0.005	41 ± 0.4	57 ± 7	1.25 ± 0.15

The NMRD profile of the gadolinium compound was fitted thanks to the Solomon-Bloembergen-Morgan (SBM) inner sphere relaxation theory with additional contributions from outer sphere [3] and second sphere relaxation [20–22]. Simple Lorentzian spectral density functions were used in the SBM equations even if for macromolecules the Lipari-Szabó spectral density functions could be more appropriate [6, 23, 24]. However, this approach—using a global rotation time, a correlation time refecting rapid local motions and a general order parameter—would add two parameters for the fitting of the NMRD profile, for a total of 8 parameters. Moreover, to confirm that the Lipari-Szabó model is adequate, additional ^17^O NMR measurements would be needed. This is beyond the scope of this communication and therefore we chose to use the Lorentzian spectral density function in the SBM equations. It is worth noting that even the SBM equations are sometimes unable to fit the low field part of NMRD profiles for slowly rotating systems [25]. Second sphere relaxation was introduced in order to take into account the contribution from water molecules of the second coordination sphere of the ion. Indeed some of the water molecules are not freely diffusing around the complex but hydrogen bonded to polar groups of the ligand. This latter relaxation term is not always easy to define but was shown to be non negligible for many Mn^2+^ and Gd^3+^ complexes. Its accurate description is difficult without further pH and temperature dependence studies of the relaxation rates. Therefore, and even if it constitutes an approximation, we used the same distance of approach (0.36 nm) for the the outer and second sphere contributions, which is a reasonable approximation for Gd^3+^ complexes [20–22]. As our introduction of the second sphere relaxation is only approximate, the number of water molecules of the second sphere contributing to relaxation (q_ss_) was not forced to be an integer. The fixed parameters of the fit were: Spin (Gd^3+^) = 3.5, number of coordinated water molecules *q* = 1, distance of closest approach for inner sphere = 0.31 nm, distance of closest approach for outer sphere and second sphere = 0.36 nm, diffusion coefficient at 25 °C = 2.3 × 10^−9^ m/s^2^. As usual for gadolinium, the scalar contribution was neglected. The fitted parameters are provided in Table 1.

The profile of iron oxide particles was fitted using the theory of Roch et al. *M*_sat_ is the saturation magnetization of the particle, *R* is the radius of the particle, *τ*_N_ is the Néel relaxation time and *p* is an empirical parameter related to the anisotropy of the crystal.

The Gadospin NMRD profile was fitted with the Solomon-Bloembergen-Morgan inner-sphere relaxation theory with additional contributions from outer-sphere and second-sphere relaxation. *τ*_R_ is the rotational correlation time of Gd^3+^ individual complexes, *τ*_M_ is the coordinated water residence time, *τ*_SO_ is the zero-field electron relaxation time, *τ*_V_ is the correlation time associated with the electron relaxation modulation, *q*_SS_ is the number of water molecules in the second sphere and *τ*_SS_ the correlation time of the interaction of Gd^3+^ with water molecules belonging to the second sphere.

The high-field data show that the relaxivities of both compounds decrease at high fields. This effect is well known—and ineluctable—for superparamagnetic particles. Such a decrease could be moved to higher field for gadolinium-based contrast agents by slowing down the rotational tumbling of the gadolinium complex. For example, this is achieved by grafting gadolinium chelates to nanoparticles and macromolecules, as dendrimers and polymers, which can even result in the appearance of a relaxivity peak at high fields [6, 26–29]. In the case of Gadospin™, the rotation of the complex is too fast (τ_R_ = 0.48 ns) to maintain high longitudinal relaxivities at high fields. This indicates the high flexibility of the gadolinium complex in the polymeric backbone, which is not beneficial in this case. From a fundamental point of view, the agreement between theories and experimental data is satisfactory. The long exchange time obtained from the fitting is compatible with previous results for pentaamide derivatives [30]. In our opinion, it is clear that the very high-field data, which are usually not included in the analysis of NMRD profiles, contains a large amount of information, and that including them in the fitting is more demanding for the theory. For example, a systematic deviation can be observed between theory and experimental results at the highest fields (300 MHz and above), for Gadospin™ which could mean that the usual Lorentzian spectral density functions are not adequate and that the Lipari-Szabó spectral density functions must be used instead. Similarly, the high-field dispersion of the NMRD profile of PEGylated superparamagnetic particles is not perfectly fitted by the Roch model [4]. This could be caused by the size distribution of the particles, which is not taken into account in this relaxation theory but was previously shown to influence relaxation [31–33]. The introduction of the influence of the particles size distribution in the NMRD fitting could be really interesting, since it would provide an estimation of the polydispersity of the particles which is crucial for biomedical applications. The development of such a model would require the whole NMRD profile, and especially the high field dispersion obtained on the shuttle device. However, even the current fitting procedure of the NMRD profile can bring interesting information about the sample: the size obtained by the NMRD fitting is 9.28 nm while the size obtained by TEM was 5.2 nm. Moreover, the magnetization provided by the fitting (Mv = 189000 A/m) is significantly smaller the bulk magnetization of magnetite (Mv = 380000 A/m). This is a clear sign of clustering of the iron oxide cores in a polymer matrix [34], which is supported by the large hydrodynamic size (30 nm) of the system. Indeed a single core with a 5 kDa PEG coating would present a smaller hydrodynamic size.

## Conclusion

NMRD profiles ranging from very low fields to very high fields were measured with a commercial fast field-cycling device and a shuttle apparatus for PEGylated superparamagnetic iron oxide particles and a polymeric gadolinium chelate. The agreement between the FFC and shuttle-based measurements at their intersection is good. The data were compared to the fit of the usual relaxation theories with a rather good agreement, even if the high-field results are not perfectly reproduced by the fitting. This shows that the complete NMRD profile should be used when trying to refine relaxation theories. Indeed they bear relevant information and are therefore more demanding for the questioned theory. Last but not least, they also allow for the direct evaluation of the efficiency of a potential contrast agent at all fields, including fields typical of clinical and small animal MRI.

## Electronic supplementary material

Supplementary material 1 (DOCX 177 kb)
