# Supplementary material for: Sample Shuttling Relaxometry of Contrast Agents: NMRD Profiles above 1 T with a Single Device
Source: Appl Magn Reson. 2016 Jan 30;47:237–46. doi: 10.1007/s00723-015-0751-7 (PMC4761365; doi:10.1007/s00723-015-0751-7)
Supplement: Supplementary file 1 — Supplementary material 1 (DOCX 177 kb) [file 723_2015_751_MOESM1_ESM.docx]

Supporting information:


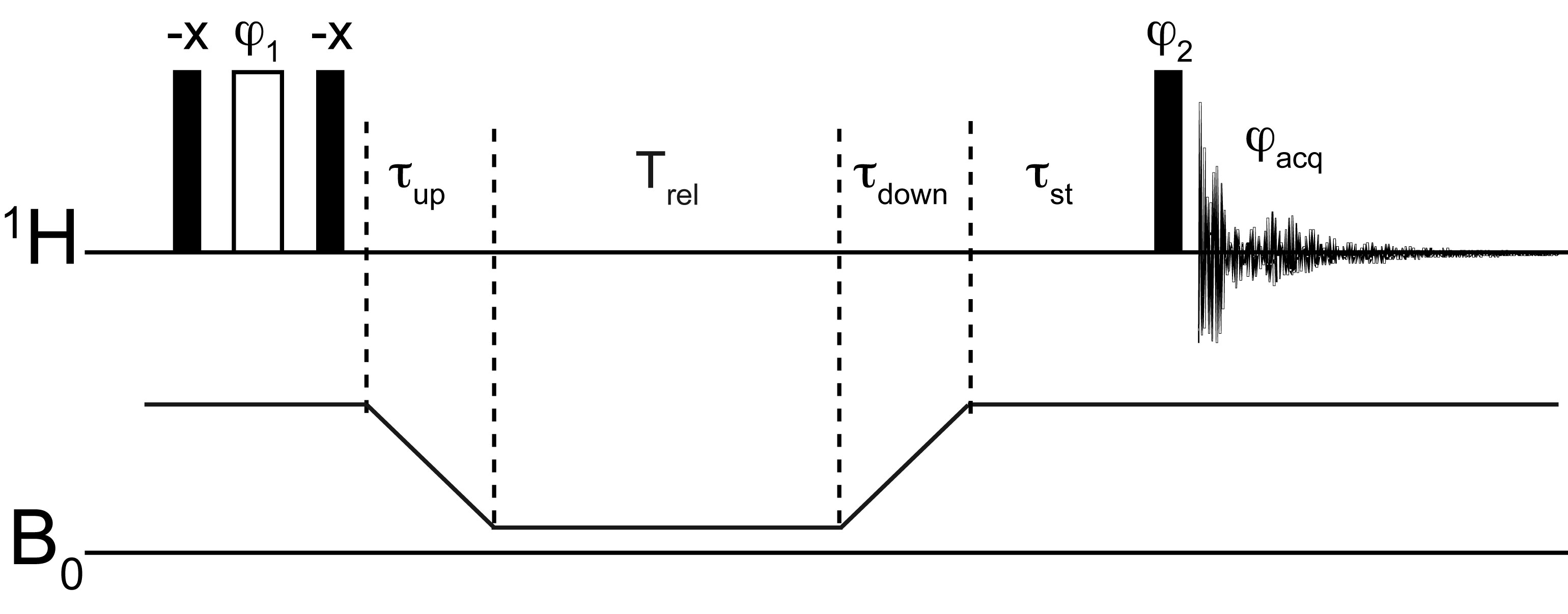


**Figure S1:** Pulse sequence used for the measurement of low-field longitudinal relaxation of water protons on the sample shuttle apparatus. Narrow filled and wide open rectangles represent 90° and 180° pulses respectively. The recycle delay was 5 s, τ_up_ and τ_down_ were between 60 and 200 ms, depending on the low-field position. The stabilization delay τ_st_ was 250 ms. The phase cycle is ϕ_1_ = {x, y}; ϕ_2_ = {x, x, -x, -x, y, y, -y, -y}; and ϕ_acq_ = {x, -x, -x, x, y, -y, -y, y}. The inversion of proton polarization every other scan leads to a decay of measured intensities (or integrals) towards zero. The delays T_rel_ were adapted to the relaxation rates at each low field. Ten different delays were chosen and two were repeated. The difference between the minimum and the maximum delays T_rel_ varied between 250 ms and 3.5 s for the USPIO solution and between 0.8 and 1 s for the Gadospin^TM^ solution.
